# Supplementary material for: New-onset IgG autoantibodies in hospitalized patients with COVID-19
Source: Nat Commun. 2021 Sep 14;12:5417. doi: 10.1038/s41467-021-25509-3 (PMC8440763; doi:10.1038/s41467-021-25509-3)
Supplement: Supplementary file 1 — Supplementary Information [file 41467_2021_25509_MOESM1_ESM.pdf]

SUPPLEMENTARY FIGURES

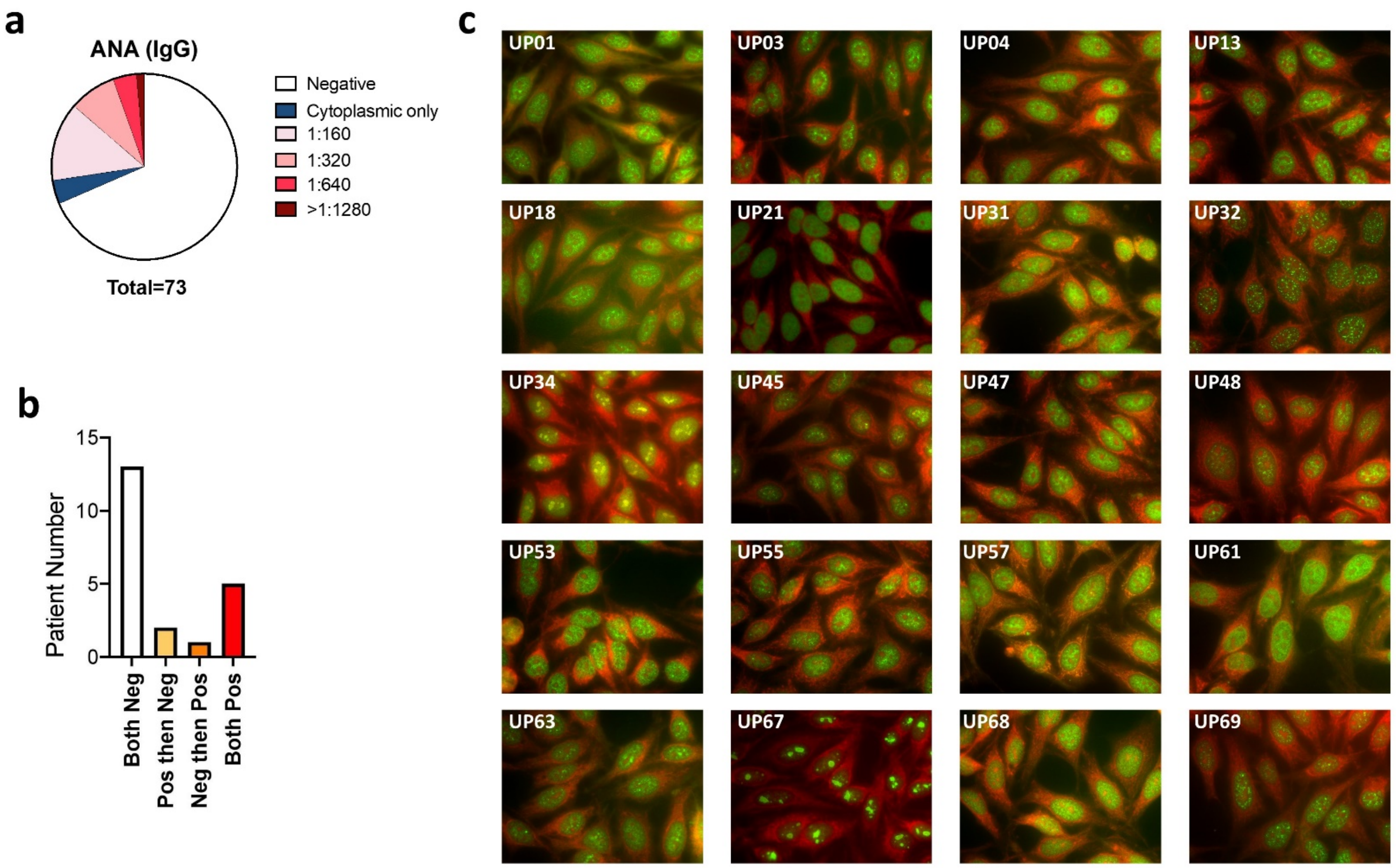

Supplementary Fig. 1: Anti-Nuclear Antibody (ANA) staining in University of Pennsylvania COVID-19 patient cohort. a IgG ANA indirect immunofluorescence results at a screening titer of 1:80, with ANA positives defined as having nuclear staining at a titer at or above 1:160. Samples with nuclear staining at 1:160 were further diluted to 1:320, 1:640 and 1:1280 to evaluate the titer. Pie chart shows patient numbers (n=73 total), color coded by ANA titers (nuclear patterns only). 3 individuals with a cytoplasmic pattern at ≥1:160 are also included as "cytoplasmic only." Further details on the staining patterns are provided in **Supplementary Table 1**. **b** Analysis of ANA data in paired samples obtained on 21 patients showing the number of patients with a change of 2-fold or more in ANA titers over time. **c** Images of positive ANAs from individual subjects. All ANA positive samples (those having nuclear staining at a titer of 1:160 or above) were imaged by fluorescence microscopy. 5 different images were collected for each sample and one representative image was chosen for display in **c**. **Supplementary**

Table 1 includes all samples that were tested in the ANA and shows which of these samples were used for imaging. Of note, UP01 was also weakly positive for dsDNA antibodies.

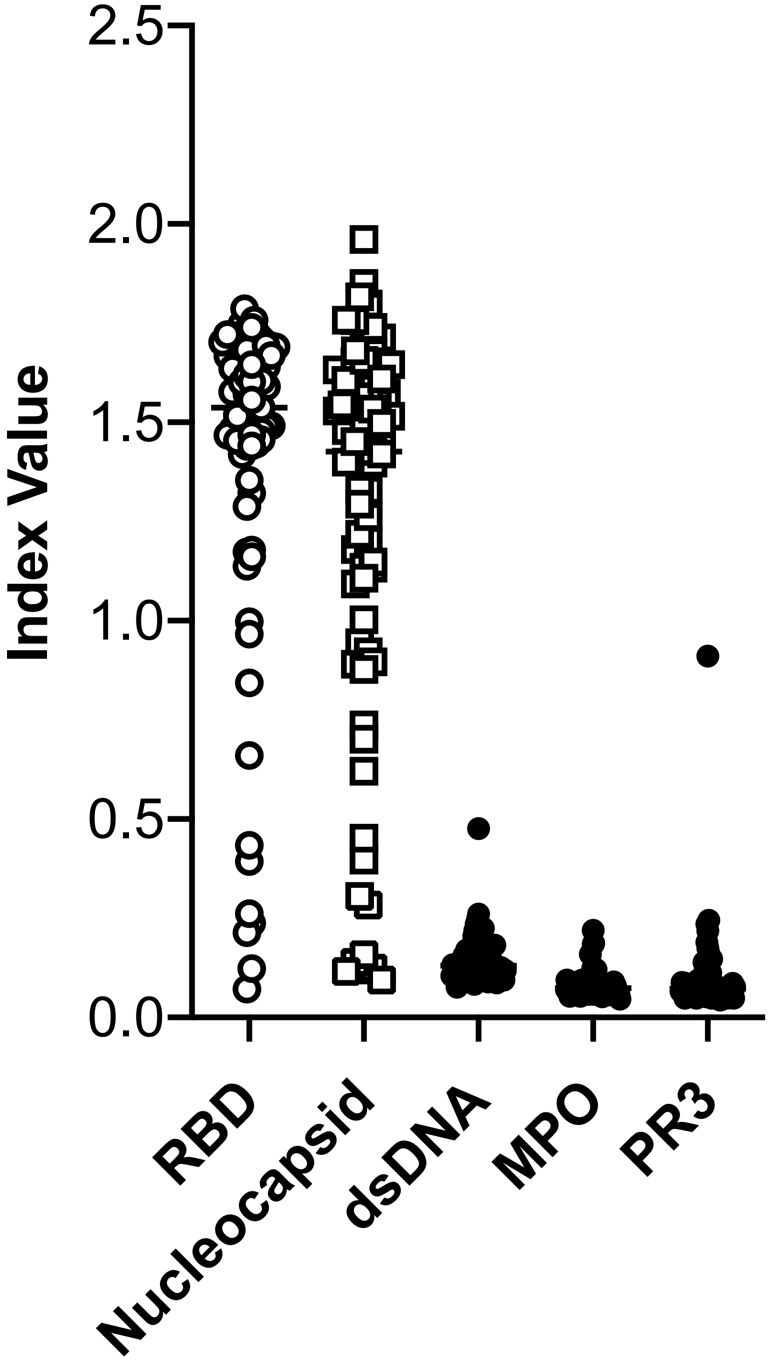

Supplementary Fig. 2: IgG ELISAs of virus and autoantigen-binding. Binding of IgG antibodies to SARS-CoV-2 receptor binding domain (RBD), nucleocapsid, and autoantigens (double stranded DNA (dsDNA), myeloperoxidase (MPO) and proteinase 3 (PR3)). Each symbol represents a patient (N=73). For subjects in which there were two or more time points, the D7 time point was chosen except for subject UP68, in whom the D14 time point was chosen. UP01 had a weakly positive dsDNA result. UP43 was positive for PR3. Source data are provided as a Source Data file.

Supplementary Figures related to Figure 1.

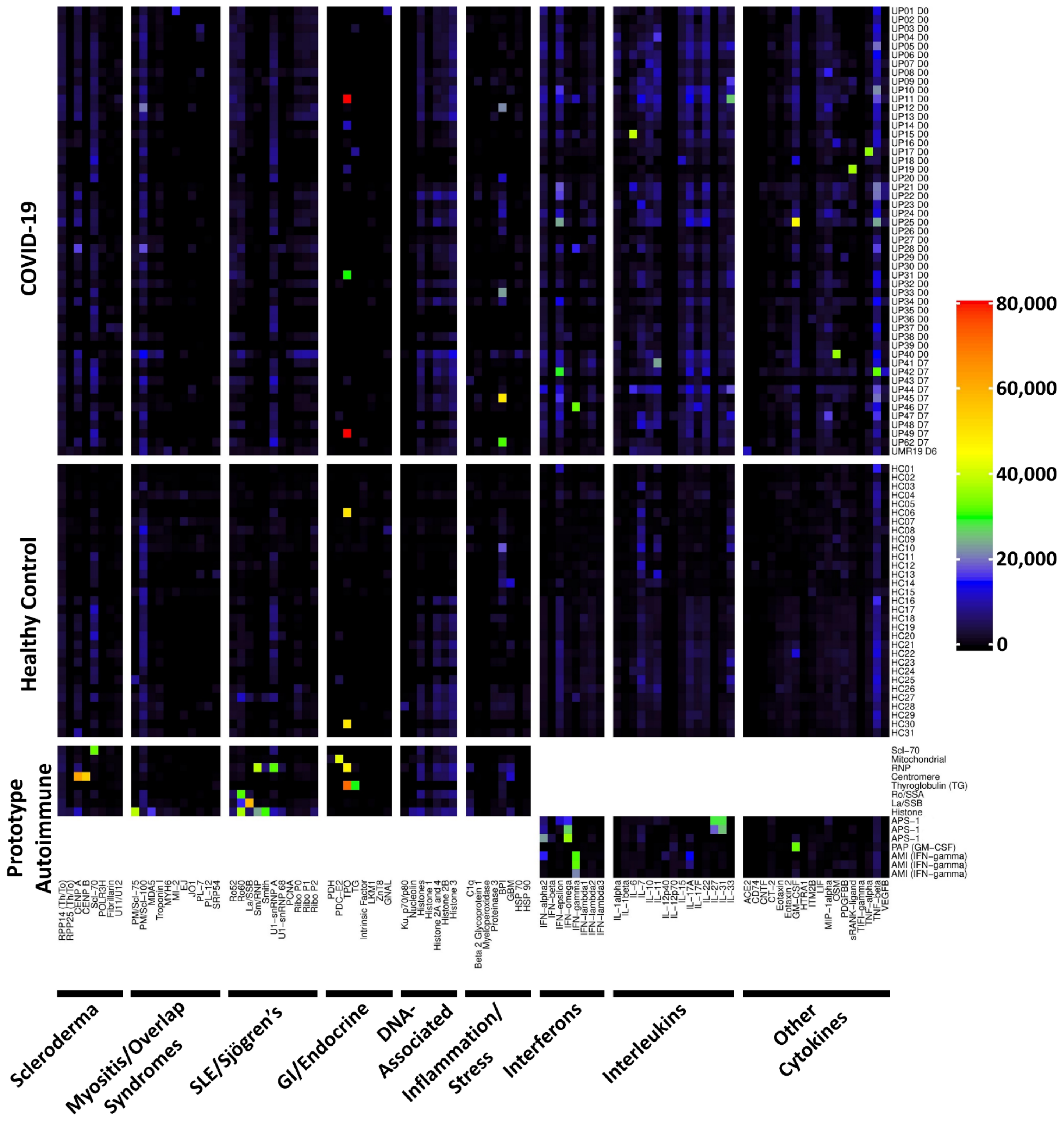

Supplementary Fig. 3: Heatmap of MFI corresponding to Figure 1. Source data are provided as a Source Data file.

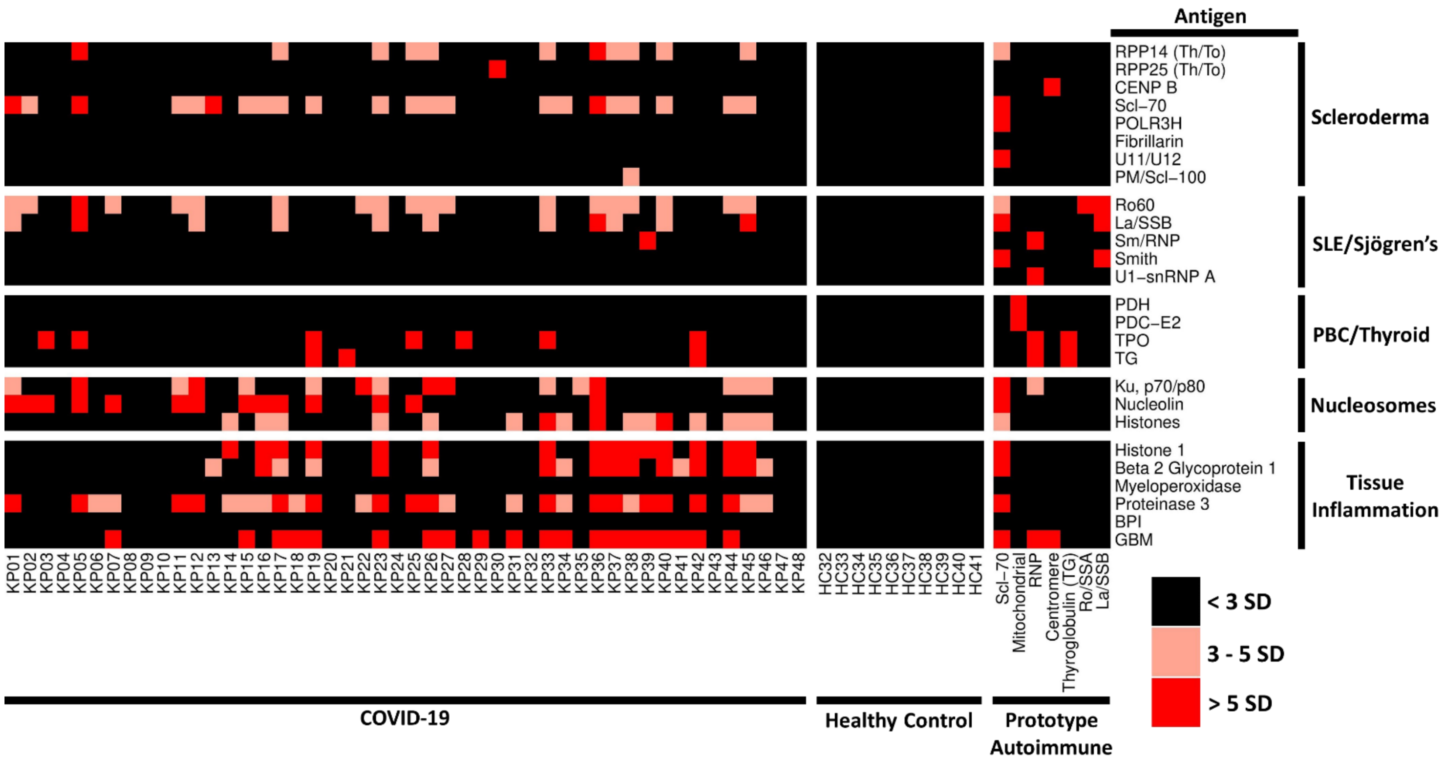

Supplementary Fig. 4: Validation in Kaiser Permanente cohort. Standard deviation heatmap depicting serum IgG antibodies discovered using a first generation 26-plex bead-based protein array containing the indicated autoantigens (y-axis). Autoantigens are grouped based on disease (scleroderma, SLE/Sjögren's, primary biliary cirrhosis (PBC)/thyroid, nucleosomes, and antigens associated with tissue inflammation. COVID-19 patients from Kaiser Permanente (left panel, n=48). HC (n=10, middle panel), and 7 prototype autoimmune disorders (right panel) are shown. Colors indicate autoantibodies whose MFI measurements are >5 SD (red), <5 or >3 SD (pink) or <3 SD (black) above the average MFI for HC. MFIs <5,000 were excluded. Source data are provided as a Source Data file.

Supplementary Figures related to Fig. 3:

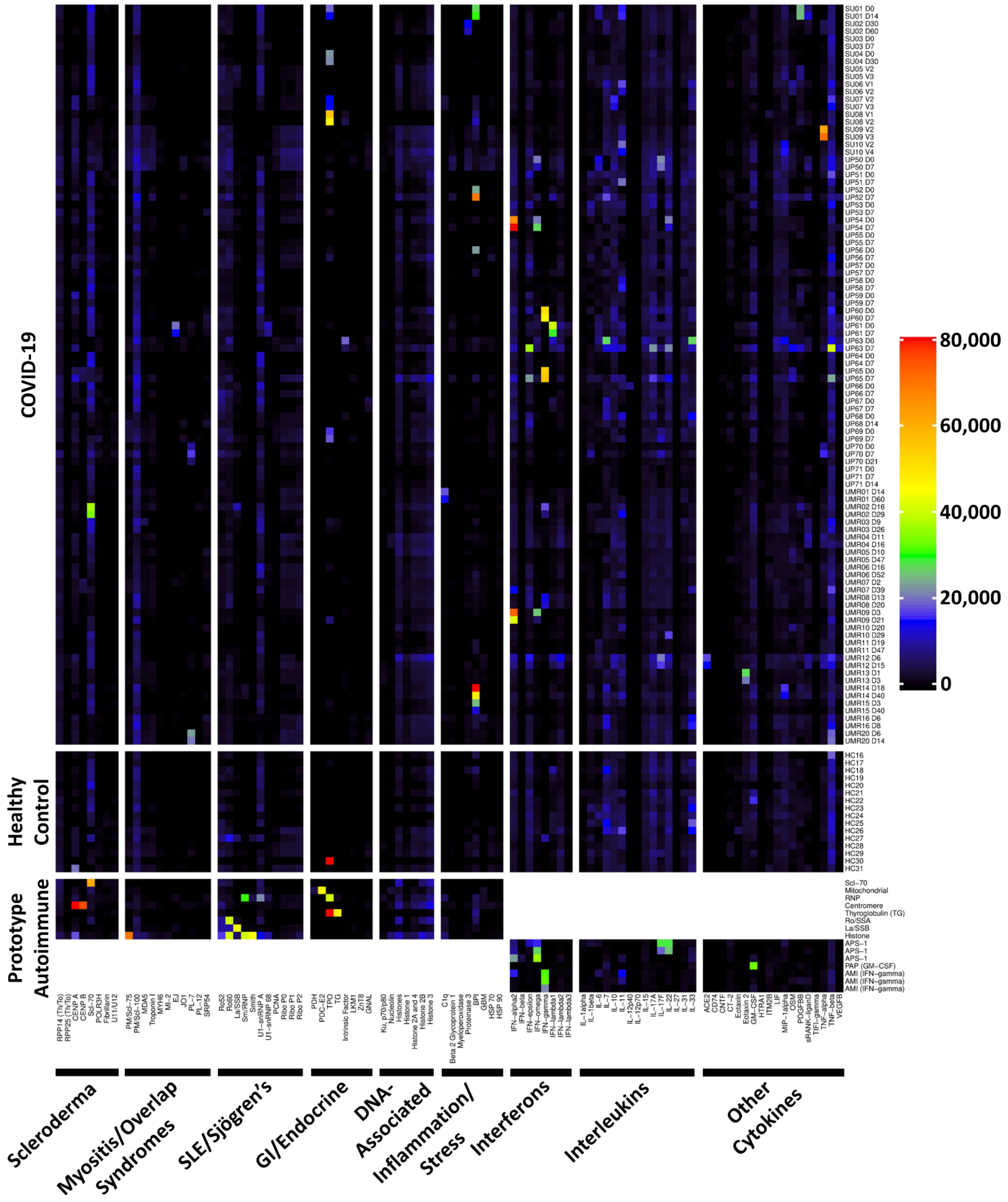

Supplementary Fig. 5: **Evolution of IgG autoantibody development over time in hospitalized COVID-19 patients. a** Heatmap using the same 53-plex bead-based protein array presented in Figure 1, containing the indicated autoantigens (x-axis). Autoantigens are grouped based on disease (scleroderma, myositis and overlap syndromes such as MCTD, SLE/Sjögren’s, gastrointestinal and endocrine disorders), DNA-associated antigens, and antigens associated with tissue inflammation or stress responses. COVID-19 patients (top panel, n=98 longitudinal COVID-19 samples, including 92 paired samples from 46 subjects and two subjects who had three available timepoints each, subject UP70 and UP71). HC (n=16, middle panel), and 8 prototype autoimmune disorders (bottom panel) are shown. **b** Heatmap using a 41-plex array of cytokines, chemokines, growth factors, and receptors. The same samples in Panel A were also analyzed for ACA. Cytokines are grouped on the x-axis by category (interferons, interleukins, and other cytokines/growth factors/receptors). Prototype samples from patients with immunodeficiency disorders include three patients with APS-1, one patient with PAP, and three patients with AMI. Colors correspond to the MFI values shown at far right. Source data are provided as a Source Data file.

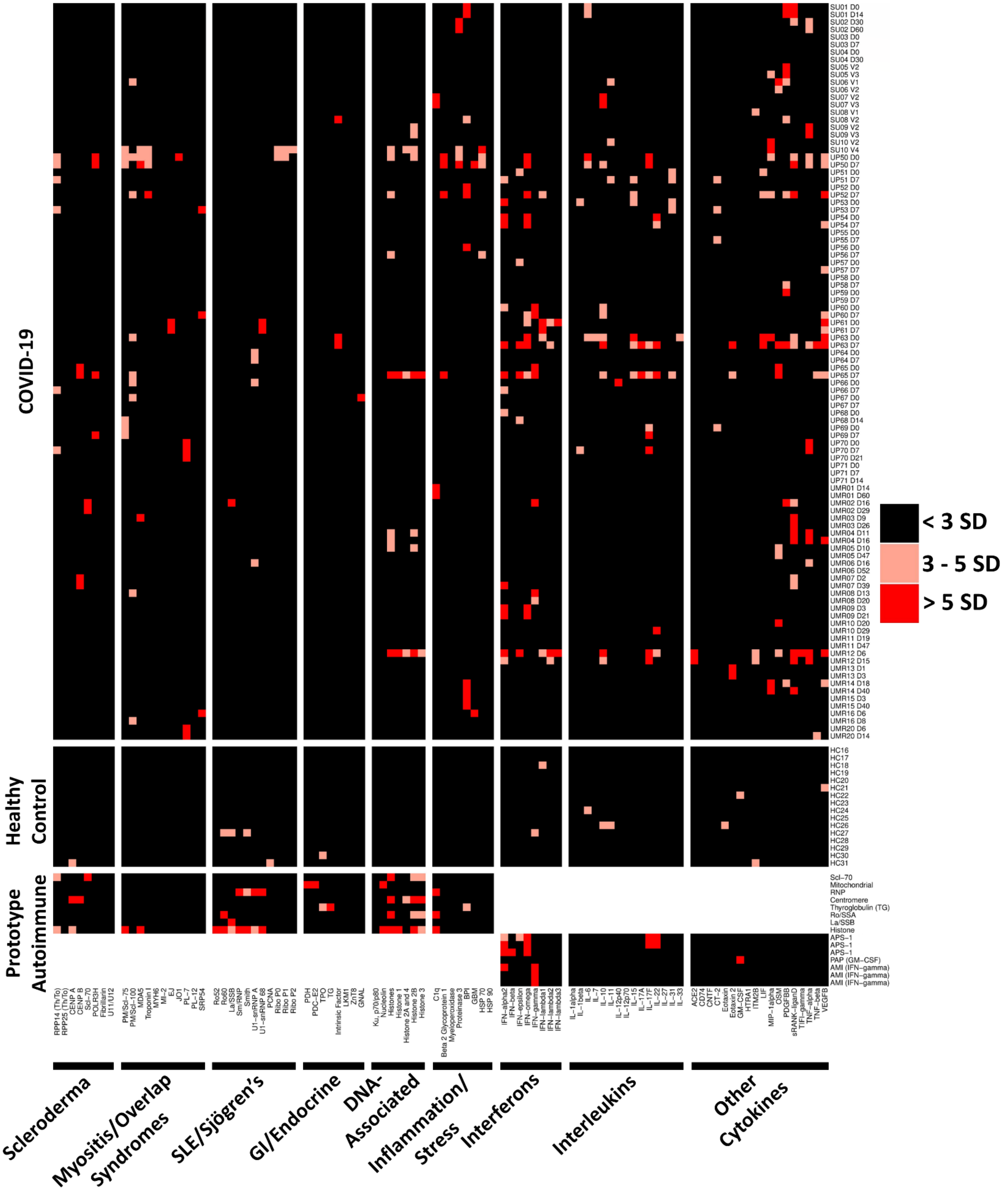

Supplementary Fig. 6: Standard deviation heatmap of MFI corresponding to Figure 3. Source data are provided as a Source Data file.

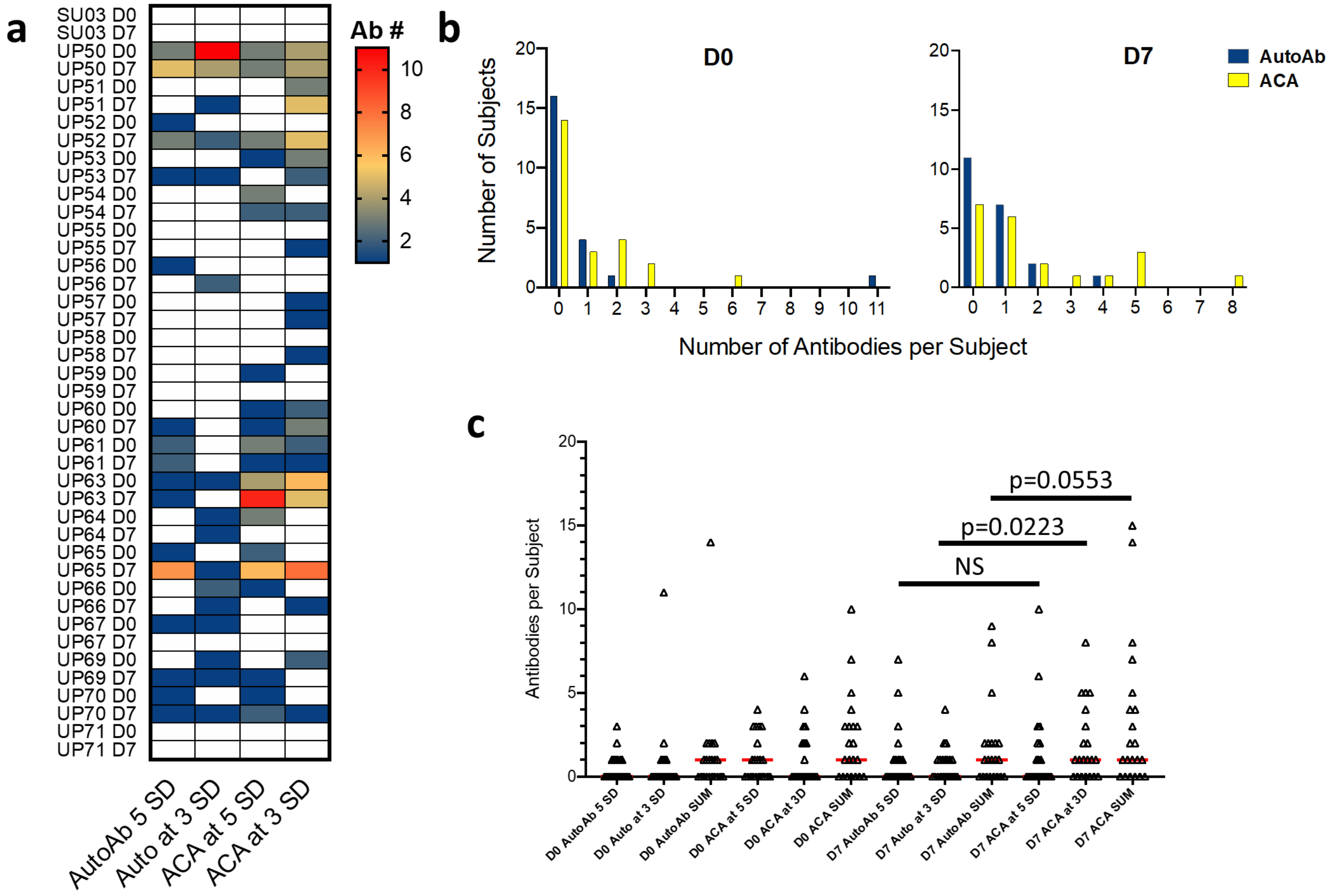

Supplementary Fig. 7: Number of antibodies in 21 subjects with paired D0 and D7 time point data, stratified by reactivity category. a Heatmap of individual subjects. Rows denote subjects and time points in days (day 0, D0 and day 7, D7). Columns denote Autoantibodies (AutoAb) and Anti-Cytokine Antibodies (ACA). Antibodies are above 5 standard deviations compared to pooled healthy controls (5 SD) or between 3 and 5 standard deviations (3 SD). White cells indicate 0 antibodies. **b** Antibody counts at D0 and D7 stratified by antibody category. Autoantibody (AutoAb, blue) and anti-cytokine antibody (ACA, yellow) counts are shown for the same 21 subjects at Day 0, (left) and Day 7 (right). Counts were based on antibodies that were present at levels between 3 and 5 SD above the average MFI for healthy control samples. **c** Antibodies per subject shown for different antibody level cut-offs. 5 SD = > 5 SD above the average MFI for the HC group; 3 SD = >3 and <5 SD above the average MFI for HC; In SUM, the 5 and 3 SD data were separately added for each individual. This

category represents all antibodies detected at 3 SD or higher. AutoAb = autoantibodies; ACA = anti-cytokine antibodies. Horizontal red lines indicate medians. Each symbol represents a subject. P-values are computed using the two-sided Wilcoxon paired rank sum test. Only D7 AutoAb vs. D7 ACA were significant ($p<0.05$) for the 3 SD and SUM antibody levels. Source data are provided as a Source Data file.

<u>Supplementary Figures related to Figs. 4 and 5:</u>

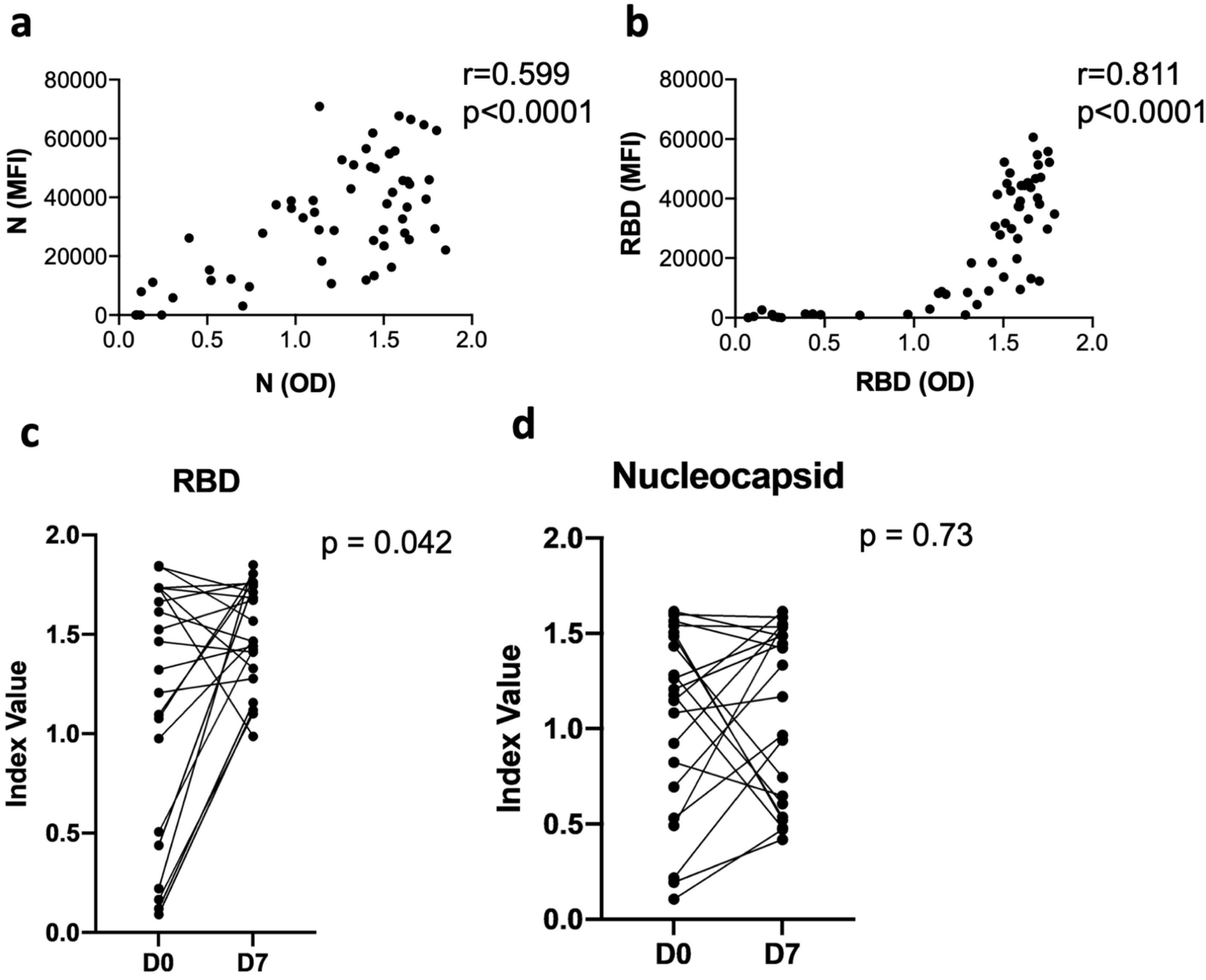

Supplementary Fig. 8: Comparison between ELISA and bead-based assays. a and b Correlation analysis between ELISA and multiplex bead data. All samples (including multiple time points from individual subjects) were included in the correlation analysis (N=57 samples from 35 individuals). Each symbol represents a sample with the mean fluorescence intensity (MFI) vs. the optical density (OD). Spearman correlations were computed with two-tailed p-values. **c and d** Two representative line plots for 21 patients with paired samples at D0 and D7. IgG antibodies increased over time for RBD (p=0.042,

two-sided Wilcoxon rank sum test) but did not change over time for Nucleocapsid (p=0.73, NS, two-sided Wilcoxon rank sum test). Source data are provided as a Source Data file.

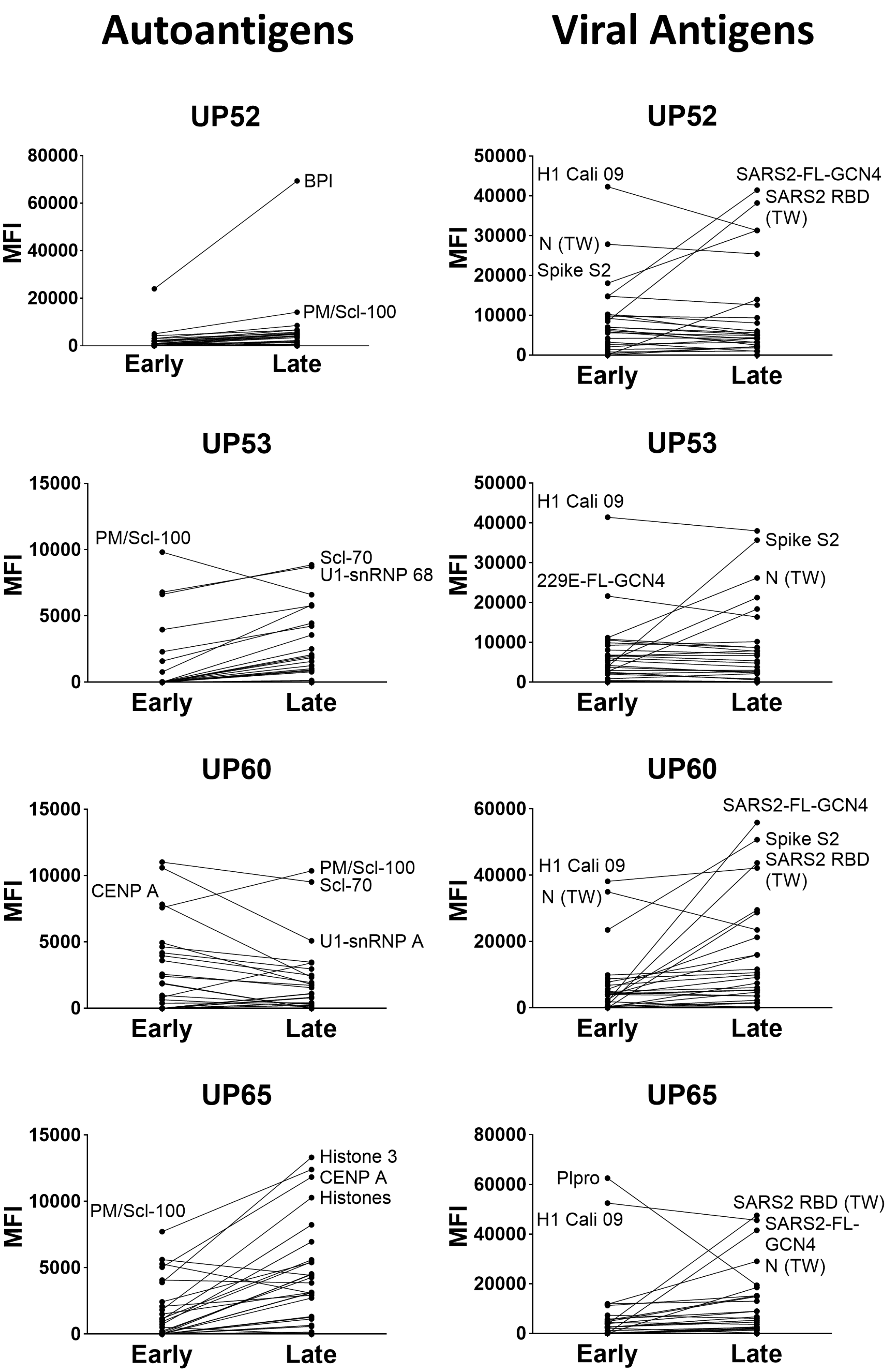

Supplementary Fig. 9: Line graphs for individual autoantigens and viral antigens for the four patients with one or more newly triggered autoantibodies, corresponding to Figure 5. Source data are provided as a Source Data file.

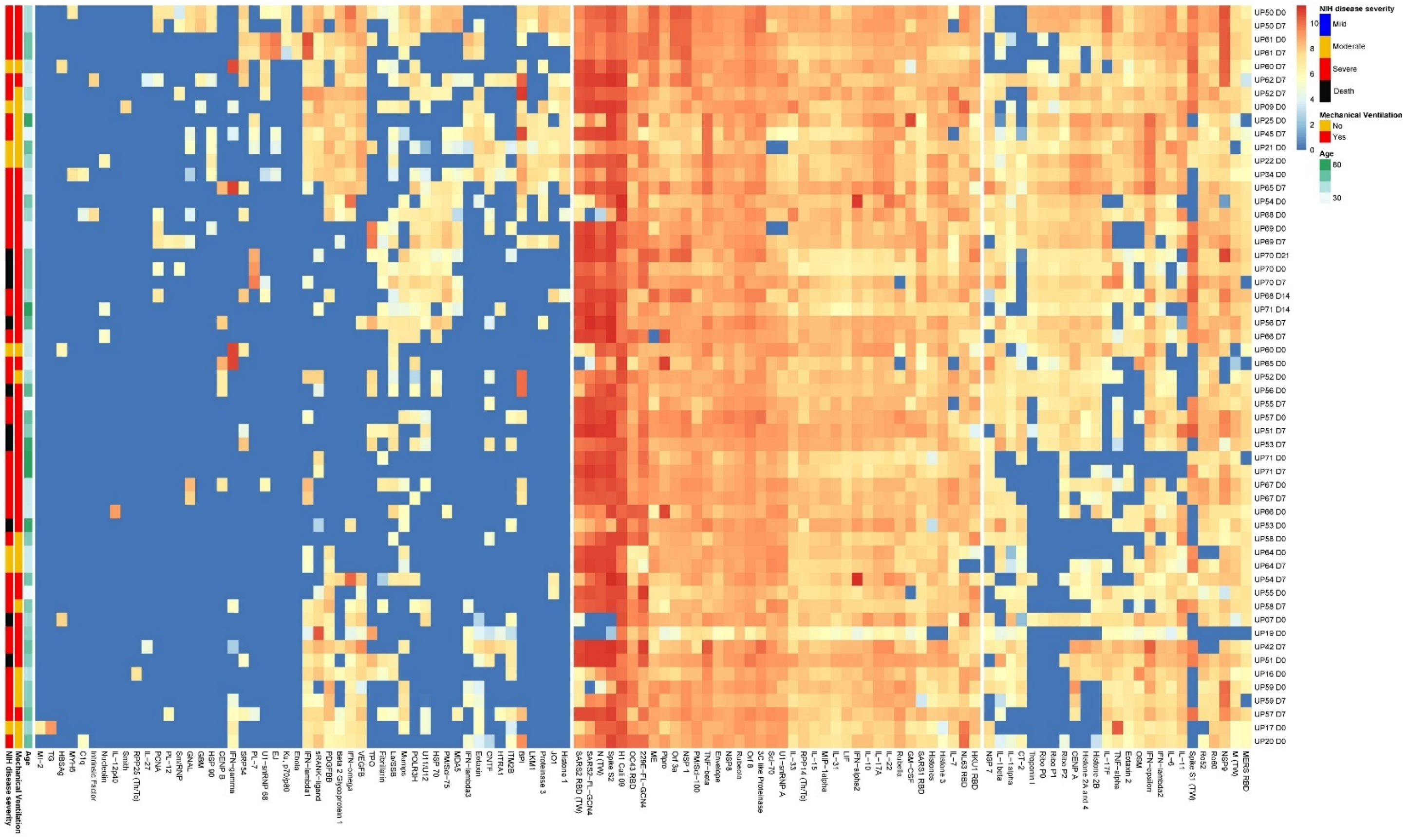

Supplementary Fig. 10: Clinical annotation of the patient cohorts enrolled at the University of Pennsylvania Health System. Heatmap demonstrating the abundance of autoantibodies and anti-cytokine antibodies (in log scale) for each subject at the timepoints indicated. To the left, clinical annotation of the patients with age, presence or absence of mechanical ventilation during hospitalization and maximum NIH disease severity score during hospitalization (levels: mild, moderate, severe, death). Source data are provided as a Source Data file.

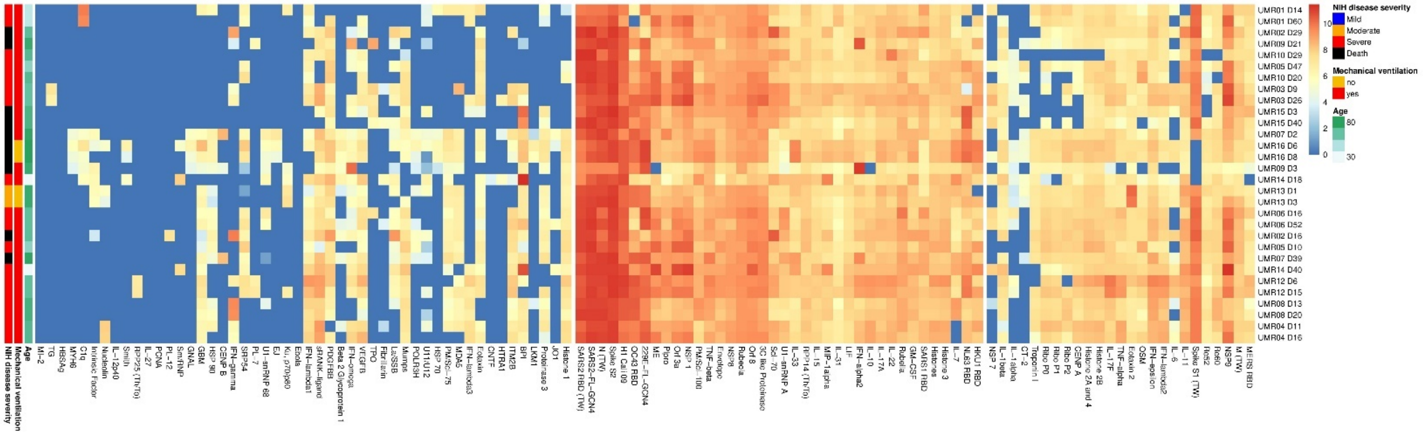

Supplementary Fig. 11: Clinical annotation of the patient cohorts enrolled at the University of Marburg. Heatmap demonstrating the abundance of autoantibodies and anti-cytokine antibodies (in log scale) for each subject at the timepoints indicated. To the left, clinical annotation of the patients with age, presence or absence of mechanical ventilation during hospitalization and maximum NIH disease severity score during hospitalization (levels: mild, moderate, severe, death). Source data are provided as a Source Data file.

SUPPLEMENTARY TABLES

Supplementary Table 1. ANA pattern and ANA titer for each UPenn sample. A dilution of 1:160 was used. This is the dilution at which ANAs are considered to be positive in the clinical lab assay, which uses the same assay kit. ANA positive samples were further diluted at 1:320, 1:640, and 1:1280 to determine the titers. Images are also provided as indicated.

ID	Timepoint (D0, D7 etc.)	Nuclear pattern	ANA Titer	Other Pattern	ANA Interpretation	Image Provided
UP01 D0	D0	Speckled	1:320	None	pos	Yes
UP02 D0	D0	None	Neg	None	neg	-
UP03 D0	D0	Speckled	1:160	None	pos	Yes
UP04 D0	D0	Speckled	1:160	None	pos	Yes
UP05 D0	D0	None	Neg	None	neg	-
UP06 D0	D0	None	Neg	None	neg	-
UP07 D0	D0	None	Neg	None	neg	-
UP08 D0	D0	Speckled	1:80	None	neg	-
UP09 D0	D0	None	Neg	None	neg	-
UP10 D0	D0	None	Neg	None	neg	-
UP11 D0	D0	None	1:80	Very weak cytoplasmic speckled	neg	-
UP12 D0	D0	None	Neg	None	neg	-
UP13 D0	D0	Speckled	1:160	None	pos	Yes
UP14 D0	D0	None	Neg	None	neg	-
UP15 D0	D0	None	Neg	None	neg	-
UP16 D0	D0	None	1:160	Cytoplasmic fluorescence	Cytoplasmic only	-
UP17 D0	D0	None	Neg	None	neg	-
UP18 D0	D0	Diffuse	1:320	None	pos	Yes
UP19 D0	D0	None	Neg	None	neg	-
UP20 D0	D0	Speckled	1:80	None	neg	-
UP21 D0	D0	Diffuse	1:640	None	pos	Yes
UP22 D0	D0	None	Neg	None	neg	-
UP23 D0	D0	None	Neg	None	neg	-
UP24 D0	D0	None	Neg	None	neg	-
UP25 D0	D0	None	Neg	None	neg	-
UP26 D0	D0	None	Neg	None	neg	-
UP27 D0	D0	None	Neg	None	neg	-
UP28 D0	D0	None	Neg	None	neg	-
UP29 D0	D0	None	Neg	None	neg	-
UP30 D0	D0	None	Neg	None	neg	-

UP31 D0	D0	Speckled	1:160	None	pos	Yes
UP32 D0	D0	Speckled	1:320	None	pos	Yes
UP33 D0	D0	None	Neg	None	neg	-
UP34 D0	D0	Nucleolar	1:320	None	pos	Yes
UP35 D0	D0	Nucleolar	1:80	None	neg	-
UP36 D0	D0	None	Neg	None	neg	-
UP37 D0	D0	None	Neg	None	neg	-
UP38 D0	D0	None	Neg	None	neg	-
UP39 D0	D0	None	Neg	None	neg	-
UP40 D0	D0	None	Neg	None	neg	-
UP41 D7	D7	None	1:160	Weak cytoplasmic speckled and spindle fibers	Cytoplasmic only	-
UP42 D7	D7	None	Neg	None	neg	-
UP43 D7	D7	Weak speckled	1:80	Weak cytoplasmic	neg	-
UP44 D7	D7	None	1:160	Weak cytoplasmic	Cytoplasmic only	-
UP45 D7	D7	Speckled	1:640	None	pos	Yes
UP46 D7	D7	Nucleolar	1:80	None	neg	-
UP47 D7	D7	Speckled	1:160	None	pos	Yes
UP48 D7	D7	Speckled	1:160	None	pos	Yes
UP49 D7	D7	Speckled	1:80	None	neg	-
UP50 D0	D0	None	Neg	None	neg	-
UP50 D7	D7	None	Neg	None	neg	-
UP51 D0	D0	None	Neg	None	neg	-
UP51 D7	D7	None	Neg	None	neg	-
UP52 D0	D0	Speckled	1:80	None	neg	-
UP52 D7	D7	None	Neg	None	neg	-
UP53 D0	D0	Speckled	1:160	None	pos	Yes
UP53 D7	D7	None	Neg	None	neg	-
UP54 D0	D0	None	Neg	None	neg	-
UP54 D7	D7	Weak nuclear	1:80	None	neg	-
UP55 D0	D0	None	Neg	None	neg	-
UP55 D7	D7	Speckled	1:160	None	pos	Yes
UP56 D0	D0	None	Neg	None	neg	-
UP56 D7	D7	None	Neg	None	neg	-
UP57 D0	D0	Speckled	1:320	None	pos	Yes
UP57 D7	D7	Speckled	1:320	None	pos	-
UP58 D0	D0	None	Neg	None	neg	-
UP58 D7	D7	None	Neg	None	neg	-
UP59 D0	D0	None	Neg	None	neg	-
UP59 D7	D7	None	Neg	None	neg	-
UP60 D0	D0	None	Neg	None	neg	-
UP60 D7	D7	None	Neg	None	neg	-

UP61 D0	D0	Speckled	1:160	None	pos	Yes
UP61 D7	D7	Speckled	1:160	None	pos	-
UP62 D7	D7	None	Neg	None	neg	-
UP63 D0	D0	Speckled	1:160	None	pos	Yes
UP63 D7	D7	None	Neg	None	neg	-
UP64 D0	D0	None	Neg	None	neg	-
UP64 D7	D7	None	Neg	None	neg	-
UP65 D0	D0	None	Neg	None	neg	-
UP65 D7	D7	None	Neg	None	neg	-
UP66 D0	D0	None	Neg	None	neg	-
UP66 D7	D7	None	Neg	None	neg	-
UP67 D0	D0	Nucleolar	> 1:1280	None	pos	Yes
UP67 D7	D7	Nucleolar	> 1:1280	None	pos	-
UP68 D0	D0	Speckled	1:160	None	pos	Yes
UP68 D14	D14	Speckled	1:640	None	pos	-
UP69 D0	D0	Speckled	1:160	None	pos	Yes
UP69 D7	D7	Speckled	1:320	None	pos	-
UP70 D0	D0	None	Neg	None	neg	-
UP70 D21	D21	None	Neg	None	neg	-
UP70 D7	D7	None	Neg	None	neg	-
UP71 D0	D0	None	Neg	None	neg	-
UP71 D14	D14	None	Neg	None	neg	-
UP71 D7	D7	None	Neg	None	neg	-

Supplementary Table 2. “COVID-19 Autoantigen Array” content.

Autoantigen Array			
**Bead ID**	**Antigen**	**Vendor**	**Catalog #**
1	Bare Bead		
2	Human IgG from serum	Sigma	I4506
3	Anti-Human IgG Fc fragment Specific	Jackson	109-005-008
4	Anti-Human IgG (H+L)	Jackson	109-005-003
5	Anti-Human IgG F(ab') fragment specific	Jackson	109-005-006
6	Beta 2 Glycoprotein 1	Diarect	A14901
7	Myeloperoxidase	Diarect	A18501
8	La/SSB	Diarect	A12801
9	Ro52	Diarect	A12701
10	Proteinase 3	Diarect	A18601
11	Histone 1	Immunovision	HIS-1001
12	Histone 2A and 4	Immunovision	HIS-1002
13	Histone 2B	Immunovision	HIS-1003
14	CENP B	Diarect	A12501
15	Histone 3	Immunovision	HIS-1004
16	Histones	Immunovision	HIS-1000
17	GBM	Diarect	A16801
18	C1q	Biodesign	A90150H
19	BPI	Arotec	ATB01-02
24	Fibrillarin	Prospec	ENZ-566

31	U11/U12	Origene	TP303746
38	CENP A	Diarect	A16901
39	EJ	Diarect	A11101
40	HSP 70	Stressgen	NSP-555
41	HSP 90	Stressgen	SPP-770
42	Intrinsic Factor	Diarect	A16701
43	JO1	Diarect	A12901
44	Ku, p70/p80	Diarect	A17301
45	LKM1	Diarect	A13501
46	MDA5	Diarect	A30001
47	MI-2	Diarect	A18101
48	PCNA	Diarect	A15401
49	PL-12	Diarect	A15701
50	PL-7	Diarect	A15601
51	PM/Scl-75	Diarect	A17001
52	Nucleolin	Diarect	A19701
53	Ribo P0	Diarect	A14101
54	Ribo P1	Diarect	A14201
55	PDC-E2	Diarect	A17901
56	Ribo P2	Diarect	A14301
57	SRP54	Diarect	A18401
58	PM/Scl-100	Diarect	A16001
59	POLR3H	Origene	TP310633
60	PDH	Sigma	P7032
62	Ro60	Diarect	A17401

63	RPP14 (Th/To)	Origene	TP760291
65	Scl-70	Diarect	A12401
67	Sm/RNP	Immunovision	SRC-3000
68	Smith	Immunovision	SMA-3000
70	Troponin I	Prospec	PRO-1269
73	TG	Diarect	A12201
74	GNAL	Abnova	H00002774-P01
75	MYH6	Origene	TP313673
76	TPO	Diarect	A12101
77	ZnT8		
78	U1-snRNP 68	Diarect	A13001
79	U1-snRNP A	Diarect	A13101
83	RPP25 (Th/To)	Origene	TP303538

Supplementary Table 3. "COVID-19 Cytokine Array" content.

Cytokine/Chemokine Array			
**Bead ID**	**Antigen**	**Vendor**	**Catalog #**
1	Bare Bead		
2	Human IgG from serum	Sigma	I4506
3	Anti-Human IgG Fc fragment Specific	Jackson	109-005-008
4	Anti-Human IgG (H+L)	Jackson	109-005-003
5	Anti-Human IgG F(ab') fragment specific	Jackson	109-005-006
6	CD74	Prospec	PRO-1467
7	IFN-lambda2	Peprotech	300-02K
8	IL-1alpha	Prospec	CYT-253
10	ITM2B	Elabscience	PKSH032599
26	IL-31	Prospec	CYT-625
27	IL-6	Prospec	CYT-098
29	OSM	Peprotech	300-10
30	IL-11	Prospec	CYT-214
32	IL-27	Prospec	CYT-048
33	CNTF	Prospec	CYT-272
34	CT-2	Prospec	PRO-1578
35	LIF	Peprotech	300-05
36	VEGFB	Peprotech	100-20B
37	HTRA1	R&D	2916-SE-020
38	GM-CSF	Peprotech	300-03

39	IFN-alpha2	R&D	11101-2
40	IFN-beta	Peprotech	300-02BC
41	IFN-gamma	Peprotech	300-02
42	IFN-epsilon	R&D	9667-ME-025/CF
43	IFN-lambda1	Peprotech	300-02L
44	IFN-lambda3	R&D	5259-IL-025/CF
45	IFN-omega	R&D	11395-1
46	IL-10	Peprotech	200-10
47	IL-12p40	Peprotech	200-12P40
48	IL-12p70	Peprotech	200-12
49	IL-15	Peprotech	200-15
50	IL-17F	Peprotech	200-25
51	IL-1beta	Peprotech	200-01B
52	IL-22	Peprotech	200-22
55	TNF-alpha	Peprotech	300-01A
56	TNF-beta	Peprotech	300-01B
58	ACE2	Sino Biological	10108-H05H
59	Eotaxin	Peprotech	300-21
60	Eotaxin 2	Peprotech	300-33
62	IL-17A	Peprotech	200-17
63	IL-33	Peprotech	200-33
64	IL-7	Peprotech	200-07
65	MIP-1alpha	Peprotech	300-08
67	PDGFBB	Peprotech	100-14B

68	sRANK-ligand	Peprotech	310-01C
69	TIFI-gamma	Diarect	A11001

Supplementary Table 4. "COVID-19 Viral Array" content.

Viral Array			
**Bead ID**	**Antigen**	**Vendor/Source**	**Catalog #**
1	Bare Bead		
2	Human IgG from serum	Sigma	I4506
3	Anti-Human IgG Fc fragment Specific	Jackson	109-005-008
4	Anti-Human IgG (H+L)	Jackson	109-005-003
5	Anti-Human IgG F(ab') fragment specific	Jackson	109-005-006
9	SARS2-FL-GCN4	Peter Kim Lab	
16	SARS1 RBD	Peter Kim Lab	
20	MERS RBD	Peter Kim Lab	
24	OC43 RBD	Peter Kim Lab	
25	229E-FL-GCN4	Peter Kim Lab	
31	NL63 RBD	Peter Kim Lab	
35	HKU1 RBD	Peter Kim Lab	
36	A/California/07/2009 (H1N1) HA	Peter Kim Lab	
37	Ebola glycoprotein (Ebola GP)	Peter Kim Lab	
56	SARS2 RBD (TW)	Taia Wang Lab	
57	Spike S1 (TW)	Taia Wang Lab	
58	M (TW)	Taia Wang Lab	
59	N (TW)	Taia Wang Lab	
70	HBSAg	MyBioSource	MBS142509
71	Mumps	Prospec	MMP-001

72	Rubella	Prospec	RUB-291
73	Rubeola	MyBioSource	MBS319759
74	NSP8	ProSci	97-097
75	Spike S2	ProSci	10-115
76	Orf 3a	ProSci	10-005
77	Orf 8	ProSci	10-002
78	3C like Proteinase	ProSci	10-116
79	NSP 1	ProSci	97-095
80	NSP 7	ProSci	97-096
81	Envelope	ProSci	10-112
82	ME	Sino Biological	40598-V07E
83	NSP9	Sino Biological	40619-V40E
92	Plpro	Sino Biological	40593-V07E

Supplementary Table 5. Marburg University patient cohort clinical characteristics.

	Marburg (N = 18)
**Age [Median (IQR)]**	66.50 (62.25-74.50)
**Sex [Percent; (N)]**	
female	17% (3)
male	83% (15)
**BMI [Median (IQR)]**	28.00 (26.17-33.35)
**Comorbidities [Percent; (N)]**	
diabetes	33% (6)
obesity	44% (8)
chronic cardiac disease	39% (7)
coronary heart disease	17% (3)
arterial occlusive disease	0% (0)
chronic obstructive pulmonary disease	0% (0)
asthma	6% (1)
renal insufficiency	11% (2)
rheumatic disorders	6% (1)
neurological neuromuscular disorders	39% (7)
oncological diseases	22% (4)
other immunosuppression	22% (4)
**Days since start of symptoms**	ND
**Days since COVID-19 diagnosis† [Median (IQR)]**	9.50 (6.00-15.50)
**Duration of hospital stay [Median; IQR]**	29.00; 21.50-39.75
**NIH Severity score**	
moderate/severe	33% (6)
critical	61% (11)
**Mortality**	28% (5)
**Intensive Care Unit**	78% (14)
**Mechanical ventilation**	83% (15)
**Supplemental oxygen**	33% (6)
**Therapy [Percent; (N)]**	
antibiotics	89% (16)
antivirals	28% (5)
antimycotics	11% (2)
dialysis	33% (6)
Extracorporeal membrane oxygenation	6% (1)
**Inflammatory Biomarkers‡ [Median (IQR)]**	
CRP [mg/L]	95.20 (47.95-145.68)
IL-6 [pg/ml]	95.50 (37.50-126.00)
complement consumption	
C3 [g/L]	1.40 (1.19-1.47)
C4 [g/L]	0.36 (0.31-0.57)
CH50 [U/ml]	48.00 (43.50-57.75)
AH50 [%]	108.00 (102.20-109.00)

†days since COVID19 diagnosis until first time point

‡first measurement during hospital stay

Supplementary Table 6. University of Pennsylvania patient cohort clinical characteristics.

	UPenn (N = 71)
**Age [Median (IQR)]**	57.5 (33-82)
**Sex [Percent; (N)]**	
female	48% (34)
male	52% (37)
**NIH Disease Severity score**	
day 0 (mean, range)	3 (2-5)
day 14 (mean, range)	4 (1-7)
Lowest achieved during enrollment	3 (1-5)
**Mechanical ventilation [Percent; (N)]**	48% (34)
**Thrombotic complications [Percent; (N)]**	32% (22)

Supplementary Table 7. Stanford patient cohort clinical characteristics.

	Stanford (N = 10)
**Age [Median (IQR)]**	44 (28-67)
**Sex [Percent; (N)]**	
female	60% (6)
male	40% (4)
**Days since COVID-19 diagnosis† [Median (IQR)]**	36 (8-56)
**NIH Severity score**	
moderate/severe	80% (8)
critical	20% (2)

†days since COVID19 diagnosis until first time point
