# Supplementary material for: New-onset IgG autoantibodies in hospitalized patients with COVID-19
Source: Nat Commun. 2021 Sep 14;12:5417. doi: 10.1038/s41467-021-25509-3 (PMC8440763; doi:10.1038/s41467-021-25509-3)
Supplement: Supplementary file 2 — Reporting summary [file 41467_2021_25509_MOESM2_ESM.pdf]

nature research

Corresponding author(s): Chrysanthi Skevaki
Eline T. Luning Prak
PJ Utz

Last updated by author(s): 07/25/2021

Reporting Summary

Nature Research wishes to improve the reproducibility of the work that we publish. This form provides structure for consistency and transparency in reporting. For further information on Nature Research policies, see our Editorial Policies and the Editorial Policy Checklist.

Statistics

For all statistical analyses, confirm that the following items are present in the figure legend, table legend, main text, or Methods section.

n/a	Confirmed	
☐	☒	The exact sample size (*n*) for each experimental group/condition, given as a discrete number and unit of measurement
☐	☒	A statement on whether measurements were taken from distinct samples or whether the same sample was measured repeatedly
☐	☒	The statistical test(s) used AND whether they are one- or two-sided<br>*Only common tests should be described solely by name; describe more complex techniques in the Methods section.*
☐	☒	A description of all covariates tested
☐	☒	A description of any assumptions or corrections, such as tests of normality and adjustment for multiple comparisons
☐	☒	A full description of the statistical parameters including central tendency (e.g. means) or other basic estimates (e.g. regression coefficient) AND variation (e.g. standard deviation) or associated estimates of uncertainty (e.g. confidence intervals)
☐	☒	For null hypothesis testing, the test statistic (e.g. *F*, *t*, *r*) with confidence intervals, effect sizes, degrees of freedom and *P* value noted<br>*Give P values as exact values whenever suitable.*
☒	☐	For Bayesian analysis, information on the choice of priors and Markov chain Monte Carlo settings
☒	☐	For hierarchical and complex designs, identification of the appropriate level for tests and full reporting of outcomes
☐	☒	Estimates of effect sizes (e.g. Cohen's *d*, Pearson's *r*), indicating how they were calculated

Our web collection on statistics for biologists contains articles on many of the points above.

Software and code

Policy information about availability of computer code

Data collection	Array data was collected using a Luminex FLEXMAP 3D® System with Luminex xPONENT® version 4.2 software. ANA images were acquired with a Hamamatsu ORCA-ER B&W CCD Digital Camera controlled with Metamorph V7.10.3.390 software.
Data analysis	Data analysis was conducted using RStudio Version 1.3.1093 and R for Windows Version 4.0.3 and GraphPad Prism software version 9.0.0. Complexheatmap v.2.7.7 was used for all heatmaps.

For manuscripts utilizing custom algorithms or software that are central to the research but not yet described in published literature, software must be made available to editors and reviewers. We strongly encourage code deposition in a community repository (e.g. GitHub). See the Nature Research guidelines for submitting code & software for further information.

Data

Policy information about availability of data

All manuscripts must include a data availability statement. This statement should provide the following information, where applicable:
- Accession codes, unique identifiers, or web links for publicly available datasets
- A list of figures that have associated raw data
- A description of any restrictions on data availability

All raw and normalized Luminex protein array data have been made publicly available in Gene Expression Omnibus (GEO) database with the SuperSeries accession code GSE180743 [https://www.ncbi.nlm.nih.gov/geo/query/acc.cgi?acc=GSE180743]. Clinical data other than data already shown in Supplementary Tables 10 and 11 are not available, to remain compliant with HIPAA requirements.

Field-specific reporting

Please select the one below that is the best fit for your research. If you are not sure, read the appropriate sections before making your selection.

- [x] Life sciences
- [ ] Behavioural & social sciences
- [ ] Ecological, evolutionary & environmental sciences

For a reference copy of the document with all sections, see nature.com/documents/nr-reporting-summary-flat.pdf

Life sciences study design

All studies must disclose on these points even when the disclosure is negative.

Sample size	Samples sizes were determined by availability of samples and logistical constraints and not any sample size calculations.
Data exclusions	One patient from the paired sample cohort was excluded from the viral array data analysis due to technical failure of a control antigen.
Replication	Samples were successfully run in duplicate using the Luminex assay with the exception of the aforementioned patient. One patient sample was successfully run in quadruplicate. All ANA positive samples (those having nuclear staining at a titer of 1:160 or above) were imaged by fluorescence microscopy. 5 different images were successfully collected for each sample and one representative image was chosen for display in Supplementary Figure 1c. Supplementary Table 7 includes all samples that were tested in the ANA and shows which of these samples were used for imaging. ELISAs were successfully performed once independently
Randomization	Samples from the four hospital systems were allocated into the "hospitalized COVID-19" experimental group based on patients meeting criteria for COVID-19. Healthy control samples were anonymously collected from Stanford Blood Bank and Stanford Hospital and Clinics from healthy donors before the pandemic.
Blinding	Luminex microarray experiments were run blinded. Data analyses to statistically identify autoantibody positive hits in the COVID-19 patient samples were not performed blinded because statistical tests compared MFI values between the two experimental groups and standard deviation cutoffs to determine autoantibody positive hits were calculated from healthy controls.

Reporting for specific materials, systems and methods

We require information from authors about some types of materials, experimental systems and methods used in many studies. Here, indicate whether each material, system or method listed is relevant to your study. If you are not sure if a list item applies to your research, read the appropriate section before selecting a response.

Materials & experimental systems

n/a	Involved in the study	
[ ]	[x]	Antibodies
[ ]	[x]	Eukaryotic cell lines
[x]	[ ]	Palaeontology and archaeology
[x]	[ ]	Animals and other organisms
[ ]	[x]	Human research participants
[ ]	[x]	Clinical data
[x]	[ ]	Dual use research of concern

Methods

n/a	Involved in the study	
[x]	[ ]	ChIP-seq
[x]	[ ]	Flow cytometry
[x]	[ ]	MRI-based neuroimaging

Antibodies

Antibodies used	The Anti-IgG reagents in the MPO, PR3, nucleocapsid and ANA assays are provided in commercially provided assay kits and the assays are performed according to the kit vendor instructions. The kit catalogue numbers are included in the methods section. ANAs were detected using a FITC-conjugated goat anti-human IgG antibody following vendor instructions (Inova Diagnostics, Cat # 708100). Anti-Myeloperoxidase (MPO) and Anti-Proteinase 3 (PR3) ELISAs were performed using Anti-IgG reagents from vendor kits (Inova Diagnostics, Cat# 708705 and Cat # 708700, respectively). Anti-SARS-CoV-2 ELISAs were performed using goat anti-human IgG-HRP (Jackson ImmunoResearch, Cat# 109-035-008). Anti-dsDNA ELISAs were performed using goat anti-human IgG alkaline phosphatase conjugate (Southern Biotech, Cat # 2040-04)<br><br>Luminex protein microarrays used R-phycoerythrin (R-PE) conjugated Fcγ-specific goat anti-human IgG F(ab')2 fragment (Jackson ImmunoResearch, Cat #109-116-098) secondary antibodies. Four antibodies were conjugated to beads in each Luminex panel as controls: Human IgG from serum (Sigma, Cat# I4506), Anti-Human IgG Fc fragment Specific (Jackson, Cat# 109-005-008), Anti-Human IgG (H+L) (Jackson, Cat# 109-005-003), and Anti-Human IgG F(ab') fragment specific (Jackson, Cat# 109-005-006).
Validation	The only primary antibody used in this study was an anti-His tag antibody (R&D Systems, Cat# MAB050-SP) that was run as a sample in Luminex experiments. The primary antibody was used as a control to verify conjugation of bead to antigens with His tags. Validation of the anti-His tag antibody was performed in the viral array experiment as evidenced by high reactivity, measured by MFI, towards antigens with His tags.

Eukaryotic cell lines

Policy information about cell lines

Cell line source(s)	Fixed and permeabilized Hep-2 cells affixed to glass slides (Inova Diagnostics, Cat # 708100) were used for ANAs.
Authentication	Hep-2 cells affixed to glass slides (Inova Diagnostics, Cat # 708100) were purchased as a kit from the vendor but were not independently authenticated by the authors.
Mycoplasma contamination	Hep-2 cells affixed to glass slides (Inova Diagnostics, Cat # 708100) were purchased as a kit from the vendor but were not independently tested for mycoplasma contamination by the authors. Morphological changes consistent with mycoplasma infection were not observed.
Commonly misidentified lines (See ICLAC register)	Hep-2 cells affixed to glass slides (Inova Diagnostics, Cat # 708100) were used in our study to perform ANAs by indirect immunofluorescence.

Human research participants

Policy information about studies involving human research participants

Population characteristics	83% of hospitalized COVID-19 patients from Philipps University Marburg were male, while the median age was 66.5. 52% of hospitalized COVID-19 patients from University of Pennsylvania were male, while the median age was 57.5. 60% of hospitalized COVID-19 patients from Stanford Occupational Health Clinic were female while the median age was 44. Co-morbidities and other clinical characteristics are described in the manuscript.<br><br>58% of healthy control serum donors were male, while the median age was 45.
Recruitment	Hospitalized COVID-19 patient serum or plasma samples were obtained following protocols approved by local institutional review boards (IRB) from 147 unique hospitalized subjects (n=99 unpaired; n=98 paired longitudinal samples from 48 distinct subjects). Samples were obtained from four centers in three distinct geographic areas: Northern California (Kaiser Permanente Health Care System, n=48 unpaired samples from hospitalized subjects) collected in March and April 2020; and Stanford Occupational Health Clinic, 20 paired samples from 10 unique hospitalized subjects) collected between April and June 2020; Philadelphia, Pennsylvania (University of Pennsylvania, n=50 unpaired; and 44 paired samples from 21 unique hospitalized subjects) obtained between April and June 2020; and Marburg, Germany (Philipps University Marburg, 1 unpaired; and 34 paired samples from 17 unique hospitalized subjects collected between April and June 2020).<br><br>Healthy controls serum and plasma samples from anonymous healthy controls (HC, n=41) were obtained prior to the COVID-19 pandemic from Stanford Blood Bank and Stanford Hospital and Clinics.<br><br>We have not determined any self-selection bias or other biases in patient sample selection. Patients were selected at each center based on their disease status (hospitalized with COVID-19).
Ethics oversight	All samples from cohorts at Stanford, University of Pennsylvania and Marburg University were obtained under informed consent. IRB numbers are provided here and in Methods. IRBs from Kaiser Permanente Health Care System (IRB# 55718), Stanford Occupational Health Clinic (IRB #55689), University of Pennsylvania (IRB #808542), and Philipps University Marburg (IRB# 57/20) approved collection of serum and plasma samples.

Note that full information on the approval of the study protocol must also be provided in the manuscript.

Clinical data

Policy information about clinical studies

All manuscripts should comply with the ICMJEguidelines for publication of clinical research and a completedCONSORT checklist must be included with all submissions.

Clinical trial registration	ClinicalTrialsgov-NCT04373148
Study protocol	Understanding Immunity to SARS-CoV-2, the Coronavirus Causing COVID-19. The protocol can be requested from the principle invesitgator, Dr. Paul Utz.
Data collection	Hospitalized COVID-19 patient serum or plasma samples were obtained following protocols approved by local institutional review boards (IRB) from 147 unique hospitalized subjects (n=99 unpaired; n=98 paired longitudinal samples from 48 distinct subjects). Samples were obtained from four centers in three distinct geographic areas: Northern California (Kaiser Permanente Health Care System, n=48 unpaired samples from hospitalized subjects) collected in March and April 2020; and Stanford Occupational Health Clinic, 20 paired samples from 10 unique hospitalized subjects) collected between April and June 2020; Philadelphia, Pennsylvania (University of Pennsylvania, n=50 unpaired; and 44 paired samples from 21 unique hospitalized subjects) obtained between April and June 2020; and Marburg, Germany (Philipps University Marburg, 1 unpaired; and 34 paired samples from 17 unique hospitalized subjects collected between April and June 2020).<br><br>Healthy controls serum and plasma samples from anonymous healthy controls (HC, n=41) were obtained prior to the COVID-19 pandemic from Stanford Blood Bank and Stanford Hospital and Clinics.<br><br>We have not determined any self-selection bias or other biases in patient sample selection. Patients were selected at each center

based on their disease status (hospitalized with COVID-19).

Outcomes

To test over time immunity to SARS-CoV-2, a recently identified coronavirus responsible for the 2019 world-wide outbreak known as COVID-19.
